# Prevalence and pattern of co-occurring musculoskeletal pain and its association with back-related disability among people with persistent low back pain: protocol for a systematic review and meta-analysis

**DOI:** 10.1186/s13643-017-0656-7

**Published:** 2017-12-16

**Authors:** Cecilie K. Overaas, Melker S. Johansson, Tarcisio F. de Campos, Manuela L. Ferreira, Bard Natvig, Paul J. Mork, Jan Hartvigsen

**Affiliations:** 1 Department of Sports Science and Clinical Biomechanics, University of Southern Denmark, Campusvej 55, 5230 Odense M, Denmark; 2 National Research Centre for the Working Environment, Copenhagen, Denmark; 3 Faculty of Medicine and Health Sciences, Department of Health Professions, Macquarie University, Sydney, NSW Australia; 4 Institute of Bone and Joint Research, Sydney Medical School, University of Sydney, Sydney, NSW Australia; 5 Institute for Health and Society, Department of General Practice, University of Oslo, Oslo, Norway; 6 Department of Public Health and Nursing, Norwegian University of Science and Technology, Trondheim, Norway; 7 Nordic Institute of Chiropractic and Clinical Biomechanics, Odense M, Denmark

**Keywords:** Persistent low back pain, Co-occurring musculoskeletal pain, Prevalence, Patterns, Systematic review protocol

## Abstract

**Background:**

Individuals with persistent low back pain commonly have a broad range of other health concerns including co-occurring musculoskeletal pain, which significantly affect their quality of life, symptom severity, and treatment outcomes. The purpose of this review is to get a better understanding of prevalence and patterns of co-occurring musculoskeletal pain complaints in those with persistent low back pain and its potential association with age, sex, and back-related disability as it might affect prognosis and management.

**Methods:**

This systematic review protocol has been designed according to the Preferred Reporting Items for Systematic Reviews and Meta-Analysis Protocols. We will perform a comprehensive search, with no date limit, in the following bibliographic databases: MEDLINE and Embase (via Ovid), CINAHL, and Scopus for citation tracking, based on the following domains: back pain, co-occurring musculoskeletal pain, combined with a focus group that emphasizes study design. Appropriate papers will be screened against the eligibility criteria by three reviewers independently, data extracted by two independent author pairs and disagreement resolved by consensus meetings or other reviewers if required. Assessment of methodological quality and risk of bias will be conducted using a modified version of the Risk of Bias Tool for Prevalence Studies developed by Hoy and colleagues. The overall risk of bias will be determined for each included study based on the raters’ consensus of the responses to the items in this tool. In case of sufficiently homogenous studies, meta-analysis will be performed.

**Discussion:**

Given the lack of standard terms used to define co-occurring musculoskeletal pain, the search strategy will include the broader term “back pain,” different terms for the “other co-occurring pain,” and specific study designs combined with several exclusion terms. The results of this proposed review will identify the prevalence and patterns of co-occurring musculoskeletal pain among those with persistent low back pain, which is likely to inform clinical management, research, and policy in management of musculoskeletal disorders.

**Systematic review registration:**

PROSPERO CRD42017068807

**Electronic supplementary material:**

The online version of this article (10.1186/s13643-017-0656-7) contains supplementary material, which is available to authorized users.

## Background

### Low back pain, co-occurring musculoskeletal pain and back-related disability

Musculoskeletal disorders are highly ranked among public health problems globally and is the second most common cause of years lived with disability (YLDs) [1]. Consequently, musculoskeletal disorders markedly affect individual’s social participation and quality of life. Among these conditions, low back pain causes approximately 10% of the total YLDs globally and is the most significant cause of severe long-term pain and physical disability and is strongly associated with poor self-rated general health [1–3].

Low back pain can range from shorter acute episodes to a trajectory of fluctuating episodes and, for some, severe and persistent pain [4]. Individuals with persistent low back pain commonly have a broad range of other health conditions and diseases, including co-occurring musculoskeletal pain in other body sites [5–7] and comorbidities such as sleep disorders, anxiety, and depression [8, 9]. The CUPID study, which included 12,410 workers from 18 countries, demonstrated that individuals who report musculoskeletal pain at one site are twice as likely to report pain at another anatomical site, in particular at anatomically adjacent sites and corresponding bilateral sites, when compared to people not reporting musculoskeletal pain [10]. Likewise, a large-scale study with more than 100,000 persons in USA found a significantly higher prevalence of comorbidities, musculoskeletal and neuropathic pain conditions, and greater use of pain-related medications in people with persistent low back pain compared to controls [8]. For some individuals, pain in multiple sites may be persistent over the adult lifespan [11] with severe negative impact on functional abilities and disability [12].

### Importance of this review

Although the presence of comorbidities can significantly affect health-related quality of life [13], symptom severity [13, 14], treatment outcomes [15], and prognosis [16, 17], current clinical guidelines on the management of musculoskeletal disorders usually provide recommendations focused at one region or single joint complaints [18]. One plausible reason is that, to date, research has failed in determining the prevalence and patterns of co-occurring musculoskeletal pain in persons with persistent low back pain and its association with disability. Better understanding of these can potentially guide clinicians in regard to the type, format and dosage of pain management. It could also potentially inform the design of targeted interventions for those with persistent multisite musculoskeletal pain conditions [8]. To our knowledge, no systematic review has been carried out to critically appraise and summarize the literature on the prevalence and pattern of co-occurring musculoskeletal pain among people reporting persistent low back pain and its potential association with age, sex, or back-related disability.

### Objectives

The primary objective of this systematic review is to identify the prevalence of co-occurring musculoskeletal pain among people with persistent low back pain. In addition, we will seek to establish whether there are any specific patterns of co-occurring musculoskeletal pain in people with persistent low back pain. Finally, we will investigate if there is an association between pain patterns, numbers of pain sites, age, sex, and back-related disability.

The following research questions will be addressed in this systematic review:

- What is the prevalence of co-occurring musculoskeletal pain among people with persistent low back pain?
- What are the patterns (e.g., number of co-occurring pain sites, distribution across body quadrants, combination of sites, and general pattern) of co-occurring musculoskeletal pain among people with persistent low back pain?
- Is there an association between pain patterns and/or number of pain sites and age, sex, or back-related disability?

### Methods/design

We have prepared and presented this protocol according to the Preferred Reporting Items for Systematic Reviews and Meta-Analysis Protocols (PRISMA-P 2015 Guidelines) [19], and its populated version for this journal is provided as Additional file 1. This systematic review protocol is registered in the international prospective register of systematic reviews (PROSPERO) with registration number CRD42017068807.

### Eligibility criteria

Studies will be included in this systematic review if they have met all inclusion criteria and will be excluded if they have met at least one of the exclusion criteria below.

Inclusion criteria

- Observational studies (i.e., longitudinal and cross-sectional cohort studies) from clinical primary care settings (e.g., general practice, physiotherapy, chiropractic, osteopathy) or based on cohorts of the general or working populations.
- Studies including adults (18 years or older) with persistent low back pain, i.e., pain within the anatomical region below the twelfth thoracic vertebra and the inferior gluteal fold with or without radiation to the legs with a duration of ≥ 4 weeks.
- Studies assessing co-occurring musculoskeletal pain (i.e., pain in more than one body site concurrently, or pattern of distribution of co-occurring musculoskeletal pain), number of co-occurring pain sites, distribution of pain sites across body quadrants and general pattern, in individuals with persistent low back pain.
- Peer-reviewed studies published in English, Norwegian, Danish, Swedish, German, Spanish, or Portuguese languages.

Exclusion criteria

- Studies including individuals with low back pain of specific pathological origin (e.g., fracture, tumor, inflammatory diseases, systemic diseases, infection, structural deformity).
- Studies including pregnant women.
- Randomized controlled trials.

### Search strategy

A comprehensive search for relevant studies, with no date limit or publication and language or geographic restriction, will be performed in the following bibliographic databases: MEDLINE and Embase (via Ovid), CINAHL, and Scopus for forward citation tracking. We will combine search term groups covering the following domains: back pain, co-occurring musculoskeletal pain, combined with a focus group that emphasizes study design. The search will not include the term “back-related disability,” but we will run separate analyses for those studies that have assessed this. A pilot search has been done on the search terminology to ensure its all-inclusiveness. Reference lists of retrieved articles and reviews will be scrutinized and forward citation tracking of key articles conducted in order to identify any further studies. Communication with content experts will also be used to identify any additional studies not identified in the computerized search. PROSPERO will be searched for ongoing or recently completed systematic reviews. Gray literature of epidemiological studies will be searched through the reference lists, content experts, and content websites (e.g., HUNT database [www.ntnu.edu/hunt], System for Information on Grey Literature in Europe, previously SIGL [www.opengrey.eu site]). Automated search updates will be set up in each database to ensure we include the latest hits in the field. Additional file 2 presents the search strategy designed for MEDLINE. Studies that are identified by our search strategy will be retrieved and managed using Endnote X8 (Thomson Reuters, Philadelphia, PA, USA).

### Study selection

Relevant records will be selected through a two-stage screening process by three independent reviewers (CKO, MJ, and TC). In the first stage, titles and, subsequently, abstracts will be screened with the reviewers blinded to each other’s selection. Disagreements will be discussed and resolved by a fourth independent author (BN) if necessary. The studies considered not to be relevant or that clearly do not meet the inclusion criteria will be excluded, and full-text articles of the remaining studies will be obtained. Studies involving the topic, but with uncertain relevance for this study, will be taken to the second stage for further considerations.

In the second stage, the three reviewers (CKO, MJ, and TC) will make the final selection based on screening of the articles in full text against the eligibility criteria. Citation tracking will be performed from the retrieved full-text articles and previous systematic reviews. If necessary, additional information will be sought from study authors to resolve questions about eligibility. Consensus meetings will be used to resolve any disagreement or, if required, by consulting a fourth reviewer (JH). Reasons for excluding literature will be recorded. A flowchart will be produced to facilitate transparency of the process.

### Data extraction

Data from the included articles will be extracted by two independent author pairs (CKO + MLF and MJ + JH), each pair including an experienced reviewer using a form. Disagreements will again be resolved first by discussion or if necessary by a third independent group of reviewers (TC, BN, and PJM).

Data extraction will include (1) first author, publication year, country, and language; (2) study topic, objective(s), and design; (3) time of study, mode of data collection, and type of population; (4) total sample, participation and response rate, cohort characteristics such as sex distribution, median/mean age, ethnicity, and socioeconomic status; (5) definition of low back pain; (6) presence and severity of back-related disability reports; (7) number and location of co-occurring musculoskeletal pain sites and patterns of co-occurring musculoskeletal pain and other information for assessment of risk of bias. We will attempt to contact study authors by e-mail when additional information is required, for example, due to missing data. To reduce potential errors, the data extraction form will be tested on randomly selected studies identified through the pilot search and amended accordingly.

### Assessment of methodological quality and risk of bias

Assessment of methodological quality and risk of bias of the included studies will be conducted at the study level using a modified version of the Risk of Bias Tool for Prevalence Studies (see Additional file 3). This tool was developed by Hoy et al. and found to have high inter-rater agreement [20]. We have modified it slightly for the purpose of this systematic review; item 6 was defined for low back pain only, and we left the example in item 7 open with regard to which questionnaire that was used apart from that it must have been validated. Individual items will be rated as “Yes” if the criterion is fulfilled. Otherwise, if the design of the study is not applicable or if there is insufficient information in the study to permit a judgment for a particular criterion, then it will be noted as “No.” Each study will be given an unweighted methodological score, expressing the proportion of fulfilled criteria out of the total number of relevant criteria. At the end, the overall risk of bias will be determined, not as an overall numeric rating, but based on the raters’ consensus given the responses to the preceding 10 individual items in this tool. This is consistent with the GRADE (Grades of Recommendation, Assessment, Development, and Evaluation) and Cochrane approaches [20–22]. All studies will be included in the evidence synthesis, but those with low risk of bias will be considered separately. All authors, which consist of both professors with experience in systematic reviews and risk of bias assessment and three PhD students, will be involved in the assessment of risk of bias.

### Analysis

The results of the data extraction and risk of bias assessment will be summarized in tables, separately for people seeking care in primary care, working, and general population samples. Our purpose is not to analyze the influence of different work exposures but to assess whether the patterns between working, care seeking, and general population samples are different. The proportion of participants with low back pain reporting co-occurring musculoskeletal pain at other anatomic sites as well as the reported number of co-occurring musculoskeletal pain sites will be described as prevalences. If studies are considered sufficiently homogenous, pooled proportions will be calculated for any additional musculoskeletal pain site, as well as the median number of additional sites reported by included studies. Standard errors will be calculated as the square root of the variance of the proportion, and pooling of results will be attempted using the inverse variance weighting method and random effects model. Statistical heterogeneity will be determined using the *I* ^2^ statistic. STATA will be used for pooled analysis.

Patterns of co-occurring musculoskeletal pain, in terms of the distribution of pain sites or the probability of pain in other anatomic regions, will be described when possible according to the definition of low back pain (symptom duration). In the case of sufficiently homogenous studies, the reported measures of association (e.g., odds ratio), between the pain pattern and/or number of pain sites and age, sex, and/or back-related disability, will also be pooled. Moreover, the impact of age and sex on the prevalence and pattern of co-occurring musculoskeletal pain will be described and pooled if enough studies have reported these associations. Results will be presented in figures and/or tables where appropriate.

## Discussion

In this systematic review, we expect to be able to map the prevalence of co-occurring musculoskeletal pain among people with persistent low back pain and to identify potential patterns of distribution. Furthermore, we will explore the possible associations with age, sex, and back-related disability.

Even though there has been increased focus on co-occurring musculoskeletal pain, the literature mainly deals with low back pain as a regional and/or separate complaint. It is likely that previous research has gathered and reported information on co-occurring musculoskeletal pain, but it is often not prioritized in the reporting of findings. We aim to identify, appraise, and summarize this information. This systematic review does not have a typical PICO framework because we do not seek to evaluate effect of interventions. Therefore, searching for relevant literature is challenging and identifying appropriate study designs is a focus in the search strategy. We choose not to include randomized controlled trials, because it is difficult to generalize their baseline findings to our target populations. We have decided to use the broader search terms such as “back pain” instead of “low back pain” to address this issue. As a result, however, our pilot searches have identified a substantial number of hits and to be able to narrow the search, we have included a number of exclusion terms such as surgery, emergencies, and pharmacology in the final search strategy.

To appraise the methodological quality of included studies, we have chosen to use the Risk of Bias Tool for Prevalence Studies developed by Hoy et al. [20]. This tool includes items on both internal and external validity and has been shown to have good inter-rater reliability. The senior authors in our review group have extensive experience within this research area and conduction of systematic reviews, which we also consider a strength when it comes to the critical appraisal in this study.

Persistent low back pain seems to be a stable phenomenon over the adult lifespan for some people [11]. Additionally, low back pain trajectories persist over long-term periods [4] and co-occur with other long-lasting multisite musculoskeletal pains, and this is associated with a broad range of health concerns [5–9, 11, 12, 23, 24]. Therefore, we need a clearer understanding of the prevalence and patterns of this multifaceted problem. There may be certain pain patterns among those with persistent low back pain that influence prognosis of symptoms. If these are identified, they can be used to improve the design of future clinical trials as stratifying variables. A better appreciation of the prevalence of co-occurring musculoskeletal pain among people with persistent low back pain can support researchers and policy makers in identifying priorities in healthcare. In addition, it may assist the development of health economic models that can assess economic impact of an intervention before its implementation [25].

## Additional files

Additional file 1: PRISMA-P Checklist. (DOCX 112 kb)

Additional file 2: MEDLINE Search Strategy. (DOCX 121 kb)

Additional file 3: Risk of bias tool. (DOCX 125 kb)

## Abbreviations

GRADE: Grades of Recommendation, Assessment, Development and Evaluation
HUNT: The Nord-Trøndelag Health Study
PRIMSA-P: Preferred Reporting Items for Systematic Reviews and Meta-Analysis Protocols
SIGL: System for Information on Grey Literature in Europe
YLD: Years lived with disability
