# Supplementary material for: Prevalence and pattern of co-occurring musculoskeletal pain and its association with back-related disability among people with persistent low back pain: protocol for a systematic review and meta-analysis
Source: Syst Rev. 2017 Dec 16;6:258. doi: 10.1186/s13643-017-0656-7 (PMC5732369; doi:10.1186/s13643-017-0656-7)
Supplement: Supplementary file 2 — MEDLINE Search Strategy. (DOCX 121 kb) [file 13643_2017_656_MOESM2_ESM.docx]

**Additional file 2 MEDLINE Search Strategy**

**Search strategy for MEDLINE (via Ovid) developed in collaboration with research librarian and amended for the other databases.**

**Ovid MEDLINE(R) Epub Ahead of Print, In-Process & Other Non-Indexed Citations, Ovid MEDLINE(R) Daily and Ovid MEDLINE(R) 1946 to Present (2017, July 13th) – Advanced search mode**

1 (back pain)

Back pain/ or Low back pain/ or (lumbago or backache or ((back or spine or spinal or lumbar) adj3 (pain* or complaint* or ache*))).ti,ab,kf.

2 (other musculoskeletal pain)

exp Arthralgia/ or exp Musculoskeletal Pain/ or Myalgia/ or Neck pain/ or ((musculoskeletal adj3 (pain* or complaint* or co-complaint*)) or ((accompan* or co-occur* or cooccur* or co-exist* or coexist* or associated or concomitant or concurrent or correlated* or concordan* or multisite or multi-site) adj3 (pain* or complaint*)) or other pain* or arthralgia or arthrodynia or cervicalgia or myalgia or polyarthralgia or neck ache* or neckache or ((ankle or ankles or arm or arms or buttock* or cervical spine or cervicothoracic or dorsal spine or elbow or elbows or finger or fingers or forearm or forearms or foot or feet or hand or hands or heel or heels or hip or hips or interscapular or joint or joints or knee or knees or leg or legs or limb or limbs or lower extremit* or lumbosacral or mid back or middle back or neck or pelvic or posterior trunk or shoulder or shoulders or thigh or thighs or thoracic spine or thoracolumbar or toe or toes or upper back or upper body or upper extremit* or wrist or wrists) adj5 (pain* or complaint* or ache*))).ti,ab,kf.

3 (focus)

Epidemiologic studies/ or Comorbidity/ or exp Case-control studies/ or exp Cohort studies/ or Cross-sectional studies/ or Prevalence/ or "Surveys and Questionnaires"/ or Health care surveys/ or Health impact assessment/ or Health surveys/ or Population/ or exp Population surveillance/ or Public health surveillance/ or Self report/ or Heath impact assessment/ or "Observational studies as topic"/ or epidemiology.fs. or ep.xs. or (cohort* or case-control or (cases adj3 control*) or case study or cross-sectional or observational or interview* or prevalence or prospective or retrospective or longitudinal or follow up or followup).ti,ab,kf. or questionnaire*.ab. or (Observational study or Interview).pt.

4 (exclusion)

4a (pregnancy and children)

Exp Pregnancy/ or exp Pregnancy complications/ or ((exp Child/ or Adolescent/) not Adult/) or (pregnan* or delivery or child* or schoolchild* or pediatr* or paediatr* or adolescen* or girls or boys or teenager* or youth).ti,kf.

4b (study design)

(Case reports or clinical trial* or controlled clinical trial or multicenter study or randomized controlled trial).pt. or Controlled before-after studies/ or (random* or trial or case report or controlled study).ti,kf.

4c (surgery, anesthesia)

Surgery.fs. or Exp Surgical procedures, operative/ or exp "Anesthesia and analgesia"/ or (surgery or surgical or neurosurgery or neurosurgical or decompression or replacement or fusion* or discectom* or diskectom* or endoscop* or arthroplast* or injection* or ablation or block or stimulation or epidural or radiofrequen*).ti,kf.

4d (imaging)

Exp Diagnostic imaging/ or (magnetic resonance or mr imag* or mri or fmri or tomograph* or radiograph* or echograph* or ultrasonography* or ultrasound).ti,kf.

4e (pathology)

Exp Neoplasms/ or exp Heart diseases/ or exp Osteoporosis/ or exp Arthritis, rheumatoid/ or exp Infection or (cancer* or tumor* or tumour* or metasta* or fracture* or trauma* or deformit* or injur* or osteoporosis or inflammat* or infecti* or rheuma*).ti,kf.

4f (emergency)

Exp Emergency medical services/ or Emergencies/ or exp Emergency Service, Hospital/ or (emergen* or acute).ti,kf.

4g (drug research)

Exp Pharmacology/ or exp Drug therapy/ or Drug therapy.fs.

**Final combination**

(1 and 2 and 3) not 4
